# CD24^+^/CD38^-^ as new prognostic marker for non-small cell lung cancer

**DOI:** 10.1186/2049-6958-8-65

**Published:** 2013-10-05

**Authors:** Feridoun Karimi-Busheri, Aghdass Rasouli-Nia, Victoria Zadorozhny, Habib Fakhrai

**Affiliations:** 1 Stem Cell Department, NovaRx Corporation, 6828 Nancy Ridge Drive, San Diego, USA; 2 Department of Oncology, Cross Cancer Institute, University of Alberta, Edmonton, Canada

**Keywords:** Biomarkers, CD24, CD38, Non-small cell lung cancer

## Abstract

**Background:**

Lung cancer is the leading cause of death among cancers in the world. The annual death toll due to this disease exceeds the combined deaths caused by colon, breast, prostate, and pancreatic cancers. As a result, there has been a tremendous effort to identify new biomarkers for early detection and diagnosis of lung cancer.

**Methods:**

In this study we report the results of screening a panel of eight non-small cell lung cancer (NSCLC) cell lines originating from different subtypes of lung cancer in an attempt to identify potential biomarkers unique to this disease. We used real-time polymerase chain reaction and flow cytometry techniques to analyze the expression of ALDHA1, EpCAM, CD133, CD24, and CD38 in this panel.

**Results:**

We demonstrate for the first time that the majority of NSCLC cells do not express levels of CD38 that would qualify it as a new biomarker for the disease. In contrast, we found that CD24 is over-expressed in 6 out of 8 of the cell lines. The combined CD24^+^/CD38^-/low^ phenotype was detected in 50% of the cell lines that are also positive for CD133 and EpCAM.

**Conclusions:**

We report that CD24^+^/CD38^-/low^ signature could potentially be used as a new biomarker for the early detection of NSCLC.

## Background

Despite being the number one killer among all cancers, potent biomarkers that can efficiently target a significant number of lung tumor cells are far from having any impact in prognosis and diagnostics of this malignancy. The five-year survival rate of all patients is only 15% and has not changed over the last thirty years [1]. According to an estimate in 2007, the cost of detection and treatment of lung cancer in the United States alone has been over $5 billion dollars per year [2]; absorbing 20% of Medicare’s total expenditures for cancer [3]. There is an intense effort underway globally to identify new molecular markers for Non-small cell lung cancer (NSCLC), in particular molecular biomarkers for the early detection as late stage lesions are strongly associated with high mortality [4,5]. It is expected that future genetic markers together with the current tumor, node, and metastasis classification will significantly improve the prognosis of NSCLC and influence treatment decision [6]. The emergence of the “-omics” era will likely revolutionize our approach towards the discovery of biomarkers. Genomics, epigenomics, and proteomics are among the new technologies that have identified potential next-generation biomarkers [7]. Analysis of microRNAs (miRNAs) and DNA methylation have led to the identification of many promising biomarkers that when integrated with other potential biomarkers could be used for the early detection of high risk lung cancer patients [4,8]. In a different study, proteomics analysis of NSCLC has led to the identification of two new proteins, PTRF/cavin-1 and MIF, as potential therapeutic targets [9]. The expression of aldehyde dehydrogenase A1 (ALDHA1) in tumor cells is the focus of attention both in diagnostic and therapeutic settings [10]. ALDH is an intracellular enzyme involved in metabolism of various molecules within cells such as retinoic acid, alcohol, cyclophosphamide, oxidative stress response, and aldehyde produced during lipid metabolism [11,12]. It has been reported that the enzyme is highly expressed in some of the NSCLC cell lines and also in the patient’s specimen [12,13]. Although little is known about epithelial cell adhesion molecule (EpCAM) gene expression in NSCLC, a few studies have reported the upregulation of EpCAM in NSCLC cell lines and specimens, notably in squamous cell carcinoma [14-16]. CD133, a transmembrane glycoprotein, has been reported to be one of the most representative markers of tumor initiating cells in various tumors such as glioblastoma and colorectal carcinoma [17,18]. The analysis of CD133 expression in stage I lung adenocarcinoma tumors has revealed an association with disease recurrence and led to the proposal that CD133 could be used as an independent prognostic marker [19]. In the last few years, increasing evidence has shed light on the importance of CD24 as a potent prognostic marker in breast, ovarian, NSCLC, and prostate cancers [5,20-22]. In a previous study, we have shown that expression of the cell surface protein CD38 is higher in cancer stem cells isolated from the H460 NSCLC [23,24]. This is a multifunctional enzyme involved in cell adhesion, signal transduction, and as a receptor in cells of the immune system [25]. CD38 contribution to disease progression and relapse in acute myeloid leukaemia and chronic lymphocytic leukemia is well established and the expression of the enzyme is considered an important prognostic marker in leukemia [26-28]. In the current study we have assessed the validity of some of the most discussed potential biomarkers of NSCLC, including CD38, in a panel of lung cancer cell lines in search of potent prognostic markers and signature phenotypes for NSCLC.

## Methods

### Material, cell lines, and culture media

All the cell lines (H460, A549, H661, H292, SW-900, SK-MES, H596, and H520) were purchased from the American Type Culture Collection (ATCC, Rockville, MD). Cells were cultured and grown in media according to ATCC recommendation. Dulbecco’s Modified Eagle Medium/F12 (DMEM/F12) was obtained from SAFC Biosciences (Lenexa, KS), B27 serum-free supplements and penicillin/streptomycin were purchased from Life Technologies (Carlsbad, CA), sodium bicarbonate and sodium pyruvate were obtained from VWR (West Chester, PA), basic fibroblast growth factor was purchased from Millipore Inc. (Billerica, MA). Tissue culture suspension plates and flasks were purchased from Sarstedt Inc. (Newton, NC), and BioCoat collagen I coated plates from BD Biosciences (San Jose, CA). ALDEFLUOR Assay Kit was obtained from Stem Cell Technologies Inc. (Vancouver, BC). Mouse anti-human CD24 phycoerythrin-conjugated (PE) was purchased from BD Biosciences Inc. (San Jose, CA), PE anti-human CD326 (EpCAM) and PE anti-human CD38 antibody from Biolegend (San Diego, CA), and mouse anti-human CD133/1 (AC133)-PE and CD133/2 from Miltenyi Biotec (Auburn, CA). All other chemicals were purchased from Sigma-Aldrich (St. Louis, MO) unless noted otherwise.

### Flow cytometry analysis

Enriched populations of lungospheres were analyzed by flow cytometry as described earlier [23,24]. Briefly, after trypsinization and washing the cells with medium, 1 × 10^6^ cells were passed through 0.45 μM filters to remove clumps of cells followed by washing with FACS buffer (phosphate saline buffer, 2% fetal bovine serum, and 2 mM ethylenediaminetetraacetic acid (EDTA)). Cells were centrifuged at 1,200 rpm for five minutes and the cell pellet was re-suspended in 100 μl FACS buffer containing 20 μl of CD24, CD38, or EpCAM antibodies. After incubation for 20 minutes on ice in the dark, cells were washed twice with 2 ml of FACS buffer and after the final wash they were re-suspended in 200–500 μl of FACS buffer. Cells were kept on ice/dark prior acquisition on Attune Acoustic Focusing from Applied Biosystems (Carlsbad, CA). As negative control, an isotype-matched labeled control was used for each antibody.

### Aldefluor assay

Aldefluor assay was performed according to the manufacturer instruction. Two sets of tubes were labeled as sample and control for each cell line to be tested. To the sample tube, 1 × 10^6^ cells were added and to the control tube 5 μl of diethylaminobenzaldehyde (DEAB), a specific ALDH inhibitor. Cells in the sample tube were mixed with 5 μl of activated ALDEFLUOR and 0.5 ml of the mixture was transferred to the control tube containing DEAB. Tubes were vortexed and incubated at 37°C for 30 min. Pelleted cells after centrifugation at 1000 rpm for 5 min were resuspended in 500 μl aldefluor assay buffer and analyzed on an Attune flow cytometer.

### Real-time reverse transcriptase-PCR

RNA was isolated from 5 × 10^6^ cells using Absolutely RNA Miniprep kit (Stratagene) according to manufacturer’s recommendations. The cDNA was synthesized by using Transcriptor First Strand cDNA Synthesis Kit (Roche Applied Science, Indianapolis, IN) from 0.5 μg of total RNA. RNA was incubated with anchored-oligo(dT)^18^ primer for 10 min at 65°C to denature template-primer mixture and chilled on ice. 5× reaction buffer, RNase inhibitors, 10 mM dNTPs mix and transcriptor reverse transcriptase were added to the reaction mixture and incubated at 50°C for 60 min, followed by 85°C for 5 min to inactivate reverse transcriptase and chilled on ice.

Real-time PCR was performed using LightCycler 480 and LightCylcer 480 SYBR Green I Master (Roche Applied Science). Master mixture containing cDNA, Syber Green Master and 100 μM forward and reverse primers were prepared on ice (Table 1). RT-PCR was performed at an initial denaturation of 95°C for 5 min, followed by 45 cycles of denaturation at 95°C for 10 sec, annealing at 60°C for 20 sec, and elongation at 72°C for 18 sec. To ensure that the expected PCR products were generated, melting curves were also analyzed. Relative mRNA expression levels were obtained by normalizing the amount of mRNA divided by that of GAPDH mRNA as an endogenous control in each sample.

**Table 1:** Primer used for RT-PCR

**Gene**	**Accession**	**Forward primer**	**Reverse primer**
CD24	NM_013230	CACGCAGATTTATTCCAGTGAAAC	GACCACGAAGA GACTGGCTGTT
CD38	NM_001775	TCTTGCCCAGACTGGAGAAAGG	TGGACCACATCACAGGCAGCTT
GAPDH	AF261085	ACCACAGTCCATGCCATCAC	TCCACCACCCTGTTGCTGTA

## Results

### Assurance of cell line identification

The authenticity of all eight NSCLC lines used in this study were validated by short tandem repeated DNA sequence (STR) as described earlier [23]. Briefly, DNA was extracted from the cell lines and amplified by PowerPlex 1.2 System (Promega, Madison WI) according to manufacturer instructions. The data then were analyzed on Applied Biosystems ABI Prism 310 Genetic Analyzer. The authentication of the cell lines were confirmed by the perfect match between the cell lines data and the parental cell lines released by American Type Culture Collection [23].

### Expression of ALDHA1

**Figure 1:**
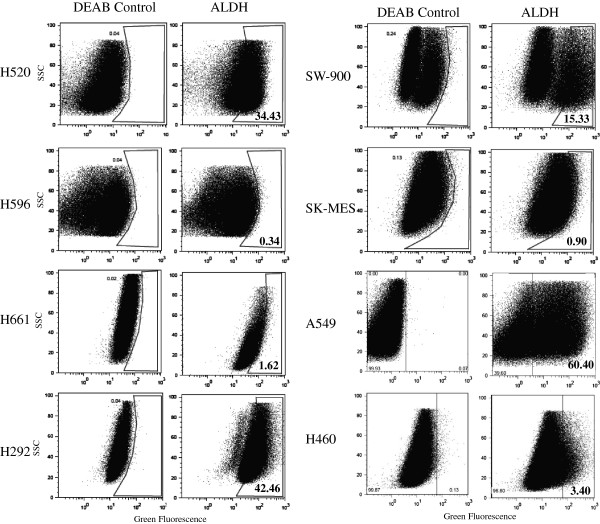
**Aldehyde dehydrogenase 1A (ALDH1) expression among the eight non-small cell lung carcinoma cell lines.** DAEB, an inhibitor of ALDH is used as negative control.

We first analyzed the expression of aldehyde dehydrogenase in eight NSCLC cell lines using Aldefluor Assay Kit (Stem Cell Technologies, Vancouver, BC) that is optimized for interaction with human ALDH 1A1. As a negative control, cells were treated with DEAB, an inhibitor of aldehyde dehydrogenase. Our analysis showed that half of the panel expresses a high level of ALDHA1 from 15.3% in SW-900, 34.4% in H520, 42.5% in H292, and 60.4% in A549. In the other four cell lines (H596, H661, SK-MES, and H460) expression was not detected or the level was very low (Figure 1). Table 2 summarizes the results of ALDH and all other markers that follow.

**Table 2:** Expression of potent biomarkers in a panel of non-small cell lung cancer cell lines

**Name**	**Type**	**ALDH**	**EpCAM**	**CD133/2**	**CD24**	**CD38**
**A549**	Adenocarcinoma	+	+	+	+	+
**H460**	Large-cell	+	+	+	+	-
**H661**	Large-cell	±	+	+	-	-
**H520**	Squamous cell carcinoma	+	+	+	+	-
**H596**	Squamous cell carcinoma	-	+	+	+	-
**SW-900**	Squamous cell carcinoma	+	+	±	+	+
**SK-MES**	Squamous cell carcinoma	±	+	+	-	-
**H292**	Mucoepidermoid pulmonary carcinoma	+	+	±	+	-

### Expression of EpCAM (CD326)

**Figure 2:**
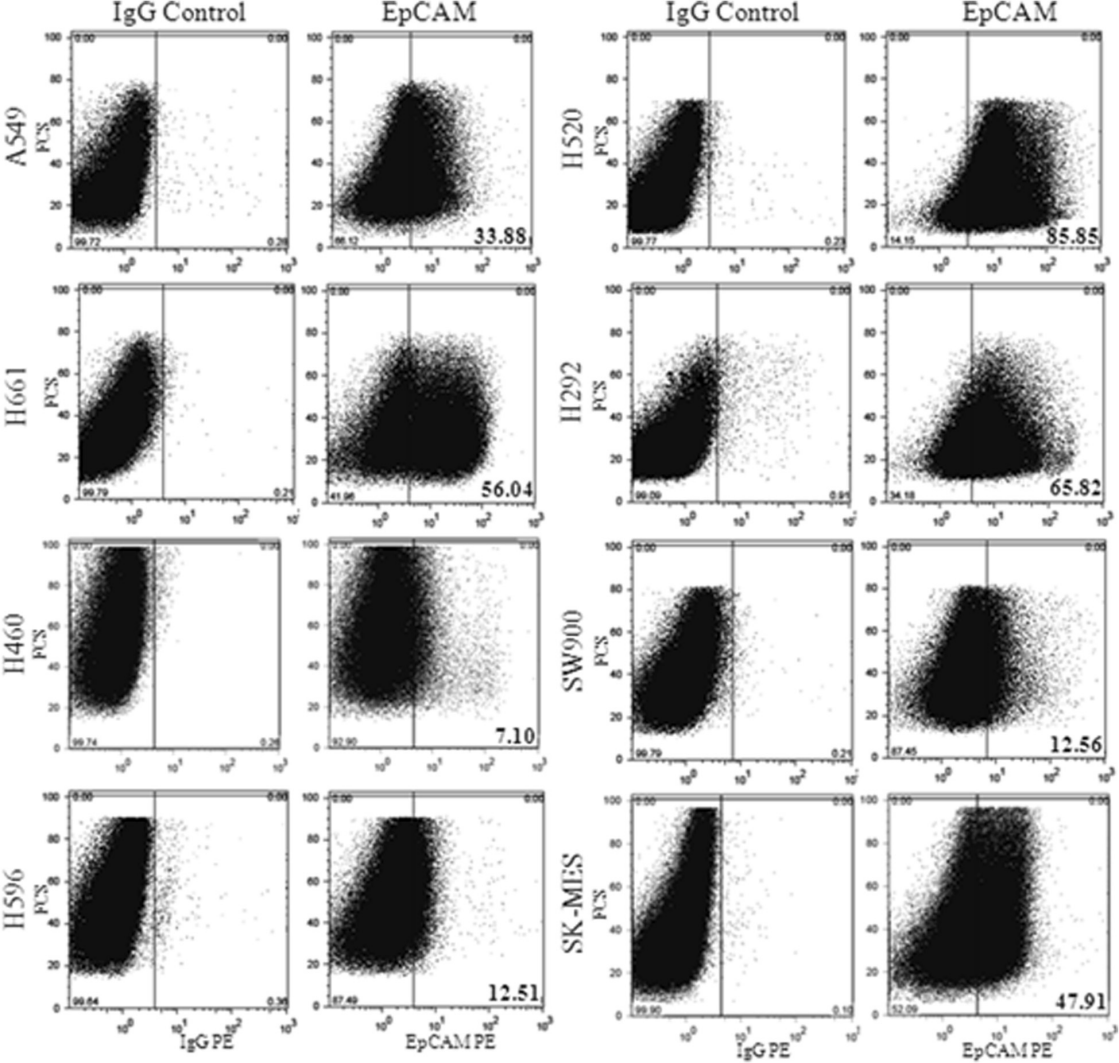
**Expression of the EpCAM (CD326) in eight non-small cell lung carcinoma cell lines as measured by flow cytometry.** IgG was used as control.

We next examined the expression of epithelial cell adhesion molecule (EpCam) in the eight cell lines (Figure 2). All the lung cancer cell lines express EpCam. However, no specific expression pattern was observed among these NSCLC based on their histology. For example, large cell carcinoma cell lines H520 and H460 display one of the highest and lowest expression among the lung panel with as high as 56.0% in H520 to 7.1% in H460. Similarly, squamous cell carcinoma cell lines express as high as 85.8% in H520 to 12.5 and 12.6% in H596 and SW900, respectively.

### Expression of CD133

**Figure 3:**
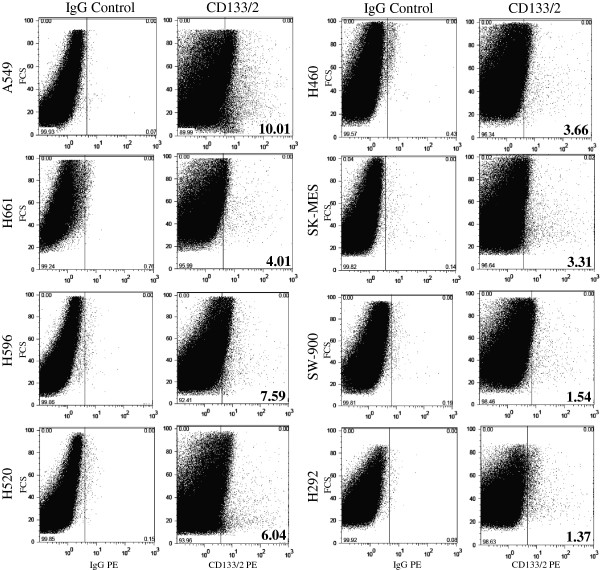
Flow cytometric analysis of CD133/2 in non-small cell lung carcinoma cell lines.

All NSCLC cell lines used in this study express a low level of CD133. The highest level was observed in A549 adenocarcinoma cell line with 10% and the lowest below 2% in SW-900 and H292 cells. The rest of the cell lines has an expression of CD133 from 3.3% in SK-MES, 3.7% in H460, 4.0% in H661, 6.0% in H520, and 7.6% in H596 (Figure 3).

### Expression of CD24 and CD38

**Figure 4:**
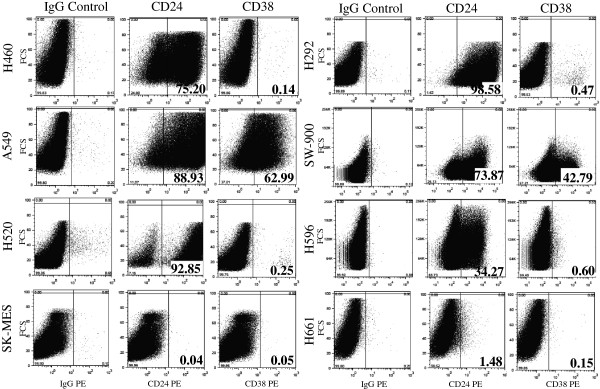
Flow cytometry analysis of the expression of CD24 and CD38 in the eight cell lines panel of non-small cell lung carcinoma.

With the exception of H661 and SK-MES cells that apparently do not express a detectable level of CD24 by flow cytometry, the expression of CD24 is very high in the other six cell lines (Figure 4). The lowest level was observed in H596 with 34.3% and the other cell lines are all above 73% and close to 100% in H292 a mucoepidermoid carcinoma cell line. In contrast the expression of CD38 is predominantly low to absent in the majority of the cell lines with the exception of A549, 63.0%, SW-900, 42.8%, and SK-MES, 35.1% (Figure 4).

**Figure 5:**
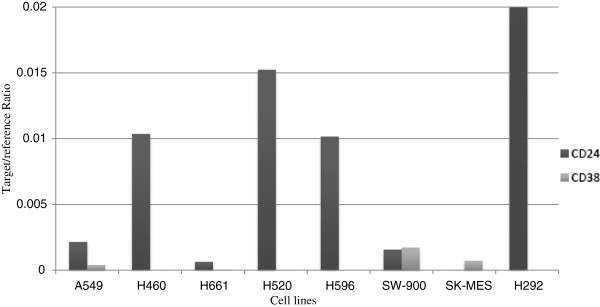
Analysis of CD24 and CD38 expression by quantitative RT-PCR in non-small cell lung carcinoma cell lines.

To validate the results of flow cytometry analysis, we extracted RNA from all the cell lines and performed a quantitative real-time PCR on the samples. As shown in Figure 5, the RNA expressions were perfectly matched with the flow cytometry results. We did not observe any RNA expression for CD24 in H661 and SK-MES cell lines, and no quantifiable CD38 RNA expression was seen in the lung cancer cell lines that were CD38^-/low^ by flow cytometry.

### Summary of the gene expression within the cell line panel

Tables 2 and 3 present an overall summary of the results in order to provide an easy reference. Table 2 displays the expression of the genes among the individual cell lines and Table 3 summarizes the percentage of the genes as potent prognostic and biomarkers for NSCLC.

**Table 3:** An overall signature of non-small cell lung cancer cell line panel and NSCLC tumor initiating cells*

**Types**	**Signature**	**%**
**Large cells**	ALDH^+^/CD38^-/low^/CD133^+^/EpCAM^+^	100
**Squamous cell carcinoma**	EpCAM^+^/CD133^+^	100
**Overall**	CD24^+^	75.0
	CD38^-/low^	75.0
	CD24^+^/CD38^-/low^	50.0
	ALDH^+^	87.50
	EpCAM^+^	100.0

* Excluding adenocarcinoma (insufficient data).

## Discussion and conclusions

In this study we provide a thorough analysis of five biomarkers of NSCLC in a panel of eight cell lines representing different types of NSCLC. Approximately 80 percent of all lung cancers are classified as non-small cell, which is further classified into three sub-types based on their morphology and physiological characteristics: squamous cell carcinoma, adenocarcinoma, and large-cell undifferentiated carcinoma. Among these sub-types, adenocarcinoma accounts for approximately 40%, followed by squamous cell carcinoma with 25-30%, and large-cell carcinoma with 10-15% [29].

The majority of information available on NSCLC is based on the available cell lines. Fortunately, scientists have access to a collection of over 200 lung cancer cell lines for their research and this has led to more than 9000 citations on the disease [30]. The results obtained from this extensive research indicate that there is a high percentage of genomic similarities between lung cancer cell lines and the tumor they have been isolated from that provides a cushion of trust for the ongoing research and the results obtained from the cell lines [30]. We are therefore confident that our findings deliver robust and reliable results that could be clinically significant for the prognosis of NSCLC patients.

A previous report on the NCI60 tumor cell line panel indicates that the expression of individual markers or combination of markers was varied among a wide range of cell lines including lung cancers [31]. One significant difference with our results, however, lies in the nature of the two panels. The NCI60 panel consists of 60 diverse human cancer cell lines widely used as a screening tool for drug discovery and representing nine distinct tumor types: leukemia, colon, lung, central nervous system, renal, melanoma, ovarian, breast and prostate [32], while our panel consists of eight cell lines specifically targeting a single malignancy, i.e., NSCLC. Our results not only confirm the presence of the variation in the expression seen among the 60 cell lines but extend the conclusion that indeed this heterogeneity and variation also exist at specific tumor derived cell lines and the three subtypes of NSCLC.

Our research shows that EpCAM is upregulated in all NSCLC cell lines. This is not surprising as it has been speculated elsewhere that proliferation, self-renewal, and invasiveness of these cells may be facilitated by the upregulation of EpCAM, leading to its use as a target of immunotherapy and treatment of human carcinoma [15,16].

Detecting EpCAM in circulating tumor cells has provoked considerable interest in cancer therapy and accordingly Food Drug Administration has set the standard for enriching circulatory tumor cells to capture and measuring the expression of EpCAM in circulating tumor cells using a magnetic ferrofluid [33]. But since EpCAM is also expressed heterogeneously in normal epithelial and in primary cells, combination therapy seems more appropriate for patients. Recently it has also reported that selected markers including EpCAM have been found to be present at high levels in the primary tumors while the level of expression was found to be low or non-detectable in normal lymph nodes or peripheral blood of NSCLC patients [34].

Our results also confirm an elevated level of activity of the ALDH in seven out of eight NSCLC cell lines where 75% of squamous cell carcinoma and all the large cells are positive for the enzyme.

CD133, a transmembrane glycoprotein, has been reported to be one of the most representative markers of tumor initiating cells and in various tumors such as glioblastoma and colorectal [17,18]. CD133 is also a marker of interest in circulatory tumor cells in malignancies including NSCLC [35]. In our study, although the entire lung cancer cell line panel expresses a low level of CD133/2, we do not see any indication that the level of expression could be an indicative of a prognostic marker in NSCLC.

We further looked at CD24 and CD38 cell surface proteins in the panel. CD24 is a potential biomarker of tumors [36] and the expression of this glycosylphosphatidylinositol-anchored receptor is upregulated in some of the cancers and in NSCLC is consistently associated with progression and metastasis of the tumors [37]. Our interest to investigate CD38 in NSCLC started with a previous finding in our laboratory that the enzyme is overexpressed in cancer stem cells isolated from a NSCLC cell line [23].

We observed an upregulation of CD24 in over 75% of NSCLC patients and for the first time we present conclusive data that 75% of the lung cell lines panel virtually do not express CD38. Previously we had also shown by immunofluorescence that the expression of CD38 is downregulated and CD24 upregulated in human lung cancer H460 cell line, one of the cell lines in current study [23]. To our knowledge this is the first report implicating an association between CD38 and NSCLC. Since a major function of CD38 is the regulation of intercellular calcium, then the absence or downregulation of the enzyme in lung cancer cell lines may indicate the disruption of intercellular calcium pathways in this disease [25,38]. If combined with CD24, 50% of the NSCLC cell lines have a CD24^+^/CD38^-/low^ phenotype that may qualify this phenotype as a new signature of NSCLC. Also of interest is the finding that all the CD24^+^/CD38^-/low^ cells are CD133, and EpCAM positive. We strongly suggest a larger scale prospective study to validate these new diagnostic biomarkers and their correlation with non-small cell lung cancer patient’s survival.

## Availability of supporting data

The data set supporting the results of the present study is present within the article.

## Competing interests

The authors declare no conflict of interests.
